# Shifting focus to preclinical stages: Locus coeruleus tau pathology as a driver and therapeutic target in Alzheimer’s disease

**DOI:** 10.4103/NRR.NRR-D-25-00140

**Published:** 2025-06-19

**Authors:** Qi Yuan, Tamunotonye Omoluabi, Brandon F. Hannam

**Affiliations:** Faculty of Medicine, Memorial University of Newfoundland, St. John’s, NL, Canada

Alzheimer’s disease (AD) remains an incurable neurodegenerative disorder with devastating societal and personal impacts. Despite decades of intensive research, therapeutic efforts targeting the clinical stages of AD have largely failed to halt or reverse disease progression. This has prompted a critical shift in focus toward the earlier, preclinical stages of AD, where interventions may hold greater promise for altering the disease trajectory. Theoretical frameworks of preclinical AD, such as those proposed by Sperling et al. (2011), describe a continuum spanning over a decade or more, characterized by three progressive stages: asymptomatic amyloidosis, the onset of neurodegeneration, and subtle cognitive impairments. While this model emphasizes amyloid-β as the initiating pathology, mounting evidence challenges this amyloid-centric paradigm, positioning early tau pathology as a primary driver of early neurodegenerative changes.

The locus coeruleus (LC), a small brainstem nucleus, is uniquely vulnerable to early tau pathology. Braak et al. (2011) identified the LC as the earliest site of hyperphosphorylated pretangle tau deposition, detectable decades before amyloid-β plaques and neurofibrillary tangles (NFTs) manifest in cortical regions. During early life, including childhood, pretangle tau accumulates in the LC and progressively spreads to other neuromodulatory nuclei and the transentorhinal cortex, likely via prion-like or transneuronal transmission, before forming NFTs in later disease stages. This trajectory underscores the critical role of LC in AD pathogenesis. Autopsy and imaging studies by Bueicheku et al. (2024) further demonstrate that LC degeneration precedes tau deposition in the medial temporal lobe and correlates strongly with early cognitive decline. Gene expression profiling has linked the selective vulnerability of LC to disruptions in protein transport regulation and mitochondrial function (Kelly et al., 2017; Bueicheku et al., 2024), accelerating its degeneration and contributing to early AD pathology.

The extensive projections of LC throughout the brain and its role as the primary site of norepinephrine (NE) production in the central nervous system underscore its importance in modulating functions such as sleep-wake regulation, attention, learning, and memory, which are often compromised in AD. Early LC dysfunction disrupts NE signaling, leading to sleep disturbance, impaired synaptic plasticity, and memory (Weinshenker, 2018). These findings position LC pretangle tau pathology as a central driver of early AD, independent of amyloid pathology. This perspective challenges traditional views of preclinical AD and suggests that targeting the LC-NE system could offer a promising strategy to modify disease progression at its earliest stages.

To investigate the role of LC pathology in preclinical AD, a novel rat model was developed by introducing human tau pseudophosphorylated at 14 sites (htauE14) into LC neurons. This model replicates key features of pretangle tau pathology, including somatodendritic tau accumulation, its spread to the neuromodulatory nuclei and entorhinal cortex, and associated cognitive impairments, all in the absence of amyloid-β plaques or NFTs (Ghosh et al., 2019; Omoluabi et al., 2021). Behavioral studies using the htauE14 model revealed deficits in challenging olfactory discrimination and spatial learning, closely mirroring the subtle cognitive impairments observed in preclinical AD. Notably, the severity of LC degeneration correlated strongly with these behavioral deficits, highlighting the central role of LC dysfunction in the early stages of AD.

The absence of amyloidosis in the htauE14 model offers a unique perspective on AD pathogenesis. These findings suggest that LC degeneration driven by pretangle tau can independently disrupt neuromodulation and cognition, potentially serving as a precursor to amyloid- and NFT-induced synaptic deficits. Mechanistic studies have revealed that hyperphosphorylated tau in LC neurons disrupts mitochondrial function, upregulates L-type calcium channel expression, and induces sex-specific transcriptomic changes, which can either exacerbate neurodegeneration or activate compensatory protective mechanisms, depending on the biological and environmental factors involved (Omoluabi et al., 2024). These findings highlight the critical importance of targeting LC pathology as a therapeutic strategy to intervene at the earliest stages of AD progression.

The LC-NE system presents a promising target for therapeutic intervention in preclinical AD. Epidemiological studies have shown that environmental factors, such as education, physical activity, and social engagement, can delay AD onset by promoting phasic LC activity (Torraville et al., 2023). In contrast, chronic stress, associated with high tonic LC firing, accelerates disease progression. An experimental study has demonstrated that phasic LC stimulation can enhance learning, reduce neuroinflammation, and protect against pretangle tau pathology (Omoluabi et al., 2021). While optogenetic techniques have been instrumental in elucidating these effects, their invasive nature limits clinical applicability, spurring interest in non-invasive approaches, such as vagus nerve stimulation (VNS), as potential therapeutic strategies.

VNS has emerged as a viable and potential non-invasive method for targeting LC function. As highlighted by Vargas-Caballero et al. (2022), VNS enhances norepinephrine release by promoting phasic LC firing, modulates neuroinflammation, improves synaptic plasticity, and enhances cognition. Beyond its potential to combat tauopathy, VNS has been shown to reduce amyloidosis and improve metabolic functioning, suggesting a multifaceted approach to AD prevention. Clinically, VNS is already approved for treating depression and epilepsy, and its efficacy in preclinical AD models further supports its translational potential. Recent advances in transcutaneous VNS have minimized side effects and surgical risks, enhancing its utility as a therapeutic tool for addressing LC dysfunction in early AD.

Furthermore, vagus nerve serves as a key link between the gut microbiota and the brain. The gut–brain axis has emerged as a promising area of interest in AD intervention. The gut microbiome plays a critical role in nervous system functions, including immune modulation, inflammation regulation, and energy metabolism. Short-chain fatty acids, such as butyrate, produced by gut microbes, enhance vagal tone, reduce systemic inflammation, and strengthen gut barrier integrity (Motataianu et al., 2023). These effects may complement the anti-inflammatory and neuromodulatory benefits of VNS, offering a synergistic approach to bolstering LC resilience and mitigating neurodegeneration.

**Figure 1:**
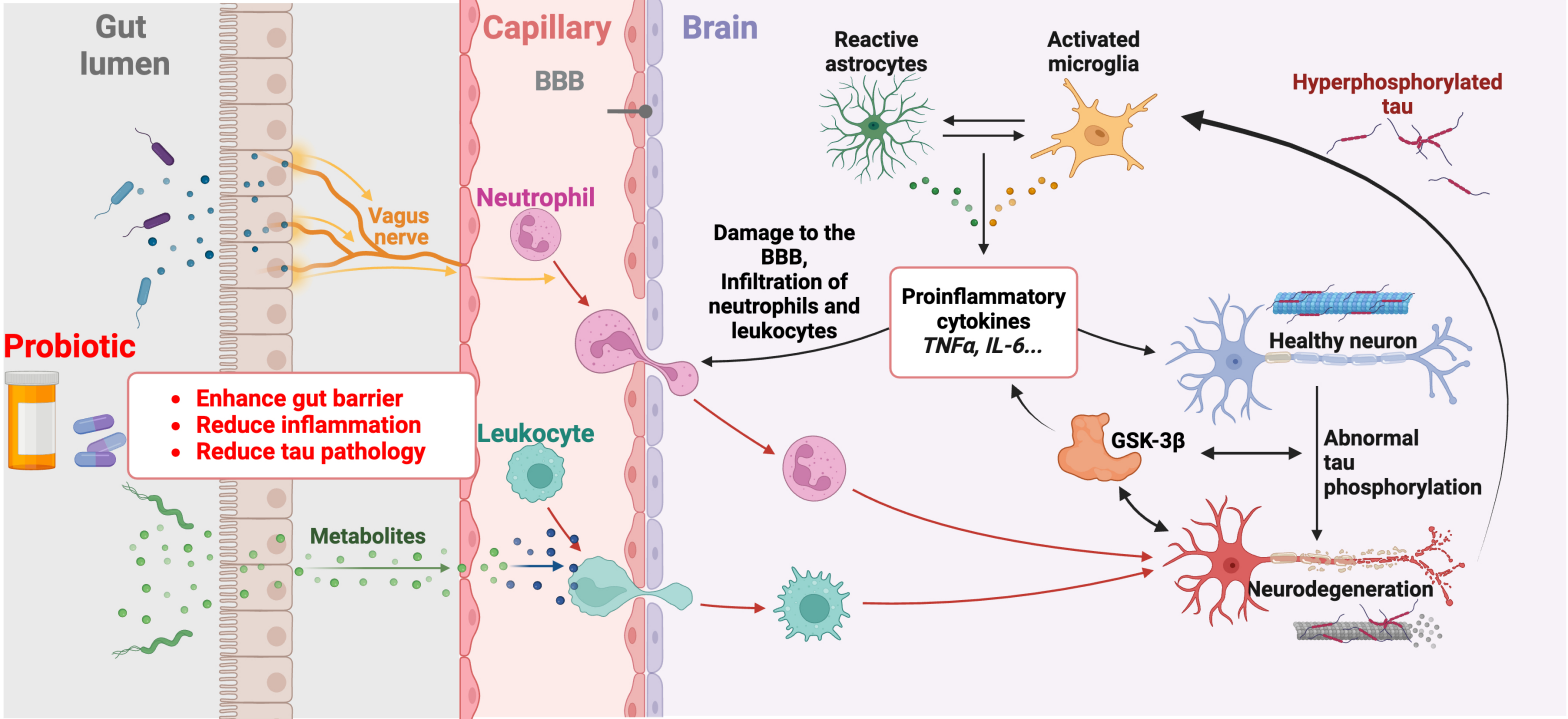
Schematic representation of hyperphosphorylated pretangle tau pathology and the therapeutic role of probiotics and vagus nerve stimulation. Probiotic supplementation enhances gut barrier integrity, promotes the production of beneficial metabolites (e.g., short-chain fatty acids such as butyrate), and reduces systemic inflammation. These effects are potentially mediated through the vagus nerve, which connects the gut to the brain, thereby influencing neuroinflammatory processes. The vagus nerve promotes anti-inflammatory pathways, modulating reactive astrocytes, activating microglia, and reducing the release of pro-inflammatory cytokines such as tumor necrosis factor alpha (TNF-α) and interleukin 6 (IL-6). This downstream effect helps prevent blood–brain barrier (BBB) damage, leukocyte infiltration, and neuroinflammation. Furthermore, probiotics and vagal signaling could attenuate glycogen synthase kinase-3β (GSK-3β) activity, a key driver of tau hyperphosphorylation, thereby protecting neurons from tau pathology and neurodegeneration. These combined mechanisms highlight a systemic approach to mitigating early Alzheimer’s disease pathology. Created with BioRender.com.

In pretangle tau models, probiotic supplementation has demonstrated the ability to positively modulate the gut microbiome, reducing neuroinflammation and improving cognitive outcomes (Flynn et al., 2025). Probiotics modulate gut-brain signaling via metabolites and vagus nerve to counteract tau pathology and neuroinflammation (**Figure 1**). Probiotics enhance gut barrier integrity, promote beneficial metabolites, and reduce systemic inflammation. These mechanisms protect the blood-brain barrier, reduce neuroinflammation, and inhibit glycogen synthase kinase-3β activity, preventing tau hyperphosphorylation and neurodegeneration, offering a systemic strategy against early AD pathology. In other animal models, the vagus nerve mediates the anxiolytic effects of probiotics (Bravo et al., 2011). When combined with VNS, probiotics could amplify the therapeutic benefits by targeting both peripheral and central mechanisms of neurodegeneration. This integrated strategy addresses the multifactorial nature of AD, potentially delaying or preventing cognitive decline by reducing LC vulnerability and enhancing neural resilience. Such interventions emphasize the importance of targeting the preclinical stages of AD, presenting a comprehensive framework for combating the early drivers of neurodegenerative processes.

Despite these advances, several critical questions remain unanswered. One of the most pressing issues is the role of sex differences in LC pathology and therapeutic efficacy. Evidence suggests that males and females may exhibit distinct patterns of LC vulnerability and tau pathology progression (Omoluabi et al., 2024), which could influence their response to interventions like VNS and probiotics. Understanding these differences is crucial for developing personalized treatments that account for sex-specific factors in disease progression and therapeutic outcomes. Furthermore, the precise mechanisms through which VNS and probiotics interact with the LC-NE system remain incompletely understood. For instance, while VNS has been shown to enhance norepinephrine release and improve cognition, the downstream molecular pathways mediating these effects in tau pathology remain elusive. Similarly, the extent to which probiotics influence central neuromodulatory systems via gut–brain signaling pathways requires further investigation. Addressing these gaps will not only optimize therapeutic strategies but also provide a deeper understanding of the interplay between peripheral and central mechanisms in AD. Large-scale longitudinal studies and advanced bioinformatics approaches will be essential to unravel these complexities and ensure the efficacy and scalability of these interventions across diverse populations.

As emerging findings challenge the traditional amyloid-centric framework of preclinical AD, LC pretangle tau pathology is increasingly recognized as a key driver of early neurodegenerative changes. Targeting the LC-NE system with innovative therapeutic strategies, such as VNS and microbiota modulation, holds the potential to reshape AD treatment by prioritizing prevention and early intervention. These approaches aim to mitigate neurodegeneration before it becomes irreversible, marking a paradigm shift in AD research and treatment. By advancing our understanding of LC pathology and developing targeted interventions, novel strategies can be devised to mitigate the progression of AD and improve outcomes for affected individuals.

*This work was supported by the Canadian Institutes of Health Research Project grant (PJT-169197) to QY. BFH was supported by a CGS-M fellowship from the Canadian Institutes of Health Research.*
